# A cloud platform for atomic pair distribution function analysis: *PDFitc*

**DOI:** 10.1107/S2053273320013066

**Published:** 2021-01-05

**Authors:** Long Yang, Elizabeth A. Culbertson, Nancy K. Thomas, Hung T. Vuong, Emil T. S. Kjær, Kirsten M. Ø. Jensen, Matthew G. Tucker, Simon J. L. Billinge

**Affiliations:** a Department of Applied Physics and Applied Mathematics, Columbia University, New York, NY 10027, USA; b Department of Chemistry, Columbia University, New York, NY 10027, USA; c Department of Chemistry and Nanoscience Center, University of Copenhagen, Copenhagen, DK 2100, Denmark; d Neutron Scattering Division, Oak Ridge National Laboratory, Oak Ridge, TN 37830, USA; e Condensed Matter Physics and Materials Science Department, Brookhaven National Laboratory, Upton, NY 11973, USA

**Keywords:** pair distribution function, PDF, data analysis, web applications, cloud computing

## Abstract

A cloud web platform for analysis and interpretation of atomic pair distribution function (PDF) data (*PDFitc*) is described. The platform is able to host applications for PDF analysis to help researchers study the local and nanoscale structure of nanostructured materials. The applications are designed to be powerful and easy to use and can, and will, be extended over time through community adoption and development. The currently available PDF analysis applications, *structureMining, spacegroupMining* and *similarity­Mapping*, are described. In the first and second the user uploads a single PDF and the application returns a list of best-fit candidate structures, and the most likely space group of the underlying structure, respectively. In the third, the user can upload a set of measured or calculated PDFs and the application returns a matrix of Pearson correlations, allowing assessment of the similarity between different data sets. *structureMining* is presented here as an example to show the easy-to-use workflow on *PDFitc*. In the future, as well as using the *PDFitc* applications for data analysis, it is hoped that the community will contribute their own codes and software to the platform.

## Abstract 2

A new web platform is presented for the pair distribution function (PDF) community to use and share advanced PDF analysis software in the cloud.

## 1. Introduction   

Much modern computing is cloud-based. When a user submits an internet search from a lightweight mobile device, it invokes a job on a high-performance computing (HPC) cluster at a remote server farm and data center. Computation is not done on the local device, but the HPC task is handled without the user even knowing. With the aid of powerful cloud computing, this job can be finished in just milliseconds (Armbrust *et al.*, 2010 ▸; Yang *et al.*, 2017 ▸; Varghese & Buyya, 2018 ▸). The trend in the whole information technology industry, not only the leading technology companies such as Google, Amazon and Microsoft, is to switch from running programs locally to cloud-based applications, yet in the physical sciences the adoption of this technology has been slower.

Deploying applications in the cloud brings a number of additional benefits beyond the ease of use and ready access to HPC resources, such as linking software to databases to allow for machine learning and recommender systems, automated software updates without user installation, and easily supporting many different operating systems and mobile devices (Kim & Korea, 2009 ▸). In the scientific research area, it seems promising to take advantage of cloud applications for data analysis programs, though this has not been widely done.

The development of a scientific technique depends on the availability of accurate, reliable, trustworthy and fast software for data analysis, and benefits from it being easy to use. For instance, in the world of structure science from diffraction data, a series of reliable data analysis programs (Larson & Von Dreele, 1994 ▸; Sheldrick, 2008 ▸; Rodríguez-Carvajal, 1993 ▸; Coelho, 2007 ▸; Altomare *et al.*, 1999 ▸) have easy-to-use graphical user interfaces (GUIs) (Toby, 2001 ▸; Toby & Von Dreele, 2013 ▸; Pape & Schneider, 2004 ▸; Roisnel & Rodríquez-Carvajal, 2001 ▸; Coelho, 2018 ▸; Farrugia, 1999 ▸), making the diffraction technique a more widely applied tool.

The atomic pair distribution function (PDF) is a diffraction technique that goes beyond just well-ordered crystals (Egami & Billinge, 2012 ▸; Billinge, 2019 ▸). It does not presume period­icity, and gives the scaled probability of finding two atoms in a material a distance *r* apart and is related to the density of atom pairs in the material. PDF analysis is an excellent tool for studying structures of many advanced materials, especially when they are nanostructured (Neder & Korsunskiy, 2005 ▸; Young & Goodwin, 2011 ▸; Terban *et al.*, 2017 ▸; Laveda *et al.*, 2018 ▸).

The current authors have also released a number of easy-to-use software packages for analyzing PDF data, such as *PDFgetX2* (Qiu *et al.*, 2004 ▸) and *PDFgetX3* (Juhás *et al.*, 2013 ▸) within *xPDFsuite* (Yang *et al.*, 2015 ▸) for PDF data processing, and *PDFfit2* within *PDFgui* (Farrow *et al.*, 2007 ▸) for structure refinements. There are some developments that have been made to provide diffraction-related calculations over the internet (Campbell *et al.*, 2006 ▸; Aroyo *et al.*, 2006*a* ▸,*b* ▸, 2011 ▸; Proffen *et al.*, 2001 ▸), though these are not designed specifically for PDF data analysis and predate cloud computing. Here we report the development of a new easy-to-use cloud-based platform called *PDF in the cloud* (*PDFitc*) for users to analyze and interpret their PDF data. It initially presents three analysis applications but more are planned in the future.

## 2. *PDFitc*   

*PDFitc* is a web-based platform that hosts applications (apps) for PDF analysis to study the local structure of nanostructured materials such as crystalline powders with disorder, nanoparticles and other nanomaterials. It is designed to be free, powerful and easy to use for chemists, materials scientists, earth scientists and anyone who needs to study the structure of materials beyond the average structure.

Beyond that, we hope that it will become a platform for the PDF community to use to share PDF tips, tricks and best practice. It will also be possible for users to ‘publish’ data sets (for example, after the accompanying manuscript has been published), thus facilitating data sharing. Over time we will incorporate new functionality in the form of new apps coming from our group. It is also our plan to be able to host apps contributed by others for the broad use of the community in such a way that, when the community uses an app, proper credit is assigned to the app developer.

Currently, it offers three useful PDF analysis applications but more will be added over time:

(i) *structureMining*: given a PDF, *structureMining* (Yang *et al.*, 2020 ▸) will discover candidate structures and return a list of them automatically, together with initial fit parameters for further analysis in structural modeling programs such as *PDFgui* (Farrow *et al.*, 2007 ▸) or *DiffPy-CMI* (Juhás *et al.*, 2015 ▸).

(ii) *spacegroupMining*: given a PDF, the app will use a pre-trained convolutional neural network to predict the most likely space group of the structure that produces the PDF (Liu *et al.*, 2019 ▸).

(iii) *similarityMapping*: given a set of two or more PDFs, it will return a plot of the Pearson product–momentum correlation matrix (Myers & Well, 2010 ▸), showing the similarity between all pairs of PDFs in the set. Users may use this to find and flag outliers in a large data set, to classify distinct PDFs into subsets, and to find things of interest such as variations in phase composition in time or space (Jacques *et al.*, 2013 ▸; Jensen *et al.*, 2015 ▸; Terban *et al.*, 2016 ▸).

The web services make use of the PDF modeling program *DiffPy-CMI* (Juhás *et al.*, 2015 ▸) and other Python packages such as *TensorFlow* (Abadi *et al.*, 2016 ▸) and *SciPy.stats* (Jones *et al.*, 2001 ▸). They are deployed on cloud computing services [currently the Google Cloud Platform (GCP)] using the Python Flask framework. This modular construction makes it easy to support more analysis apps in the future if they are written in Python or have a Python interface.

**Figure 1:**
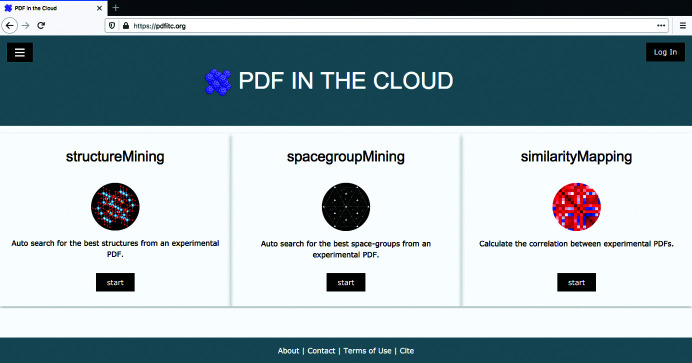
The home web page of *PDFitc*.

*PDFitc* is available at https://pdfitc.org. Its home web page is shown in Fig. 1 ▸. The user can log in using the institution identifier account through Shibboleth (Morgan *et al.*, 2004 ▸), which allows secure access to web services from a number of academic research institutions and organizations over the world. Alternatively, the user can use a Google or GitHub account authentication through OAuth to log in. More third-party OAuth apps can be supported if necessary in the future. Once logged in, the user can use the web apps. For example, to use *structureMining*, the user simply uploads a PDF and gets the answer back once the calculation finishes in the cloud, as we summarize below. More detailed instructions for using *PDFitc* are available in an ‘Instructions’ link in each app. We present *structureMining* as an illustrative example below.

## 3. *PDFitc* user interface using *structureMining* as an example   

**Figure 2:**
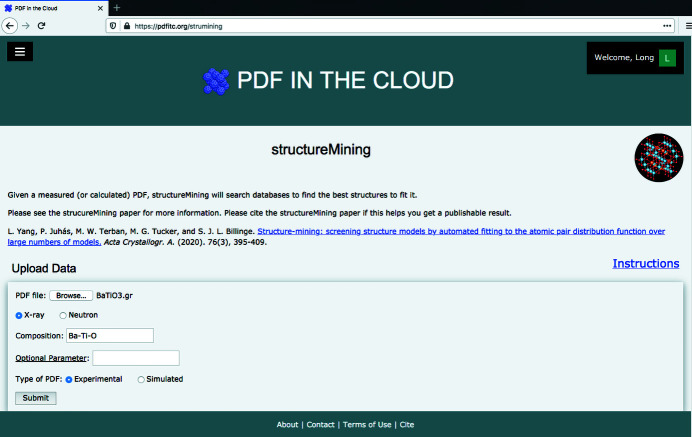
The subpage of the *structureMining* application.

Here we use *structureMining* as an example to demonstrate the easy-to-use workflow on *PDFitc* for analyzing PDF data. After login, the user clicks on the app of choice to go to the application subpage that is shown in Fig. 2 ▸ for *structureMining*.

If users are familiar with the interface they can simply browse for a .gr or similar text-format file containing a PDF on their file system and upload it. Otherwise they can follow the ‘Instructions’ link to get more help.

We use an experimental X-ray PDF of barium titanate nanoparticles (Lombardi *et al.*, 2019 ▸) as an example data file here to illustrate the *structureMining@PDFitc* workflow in steps.

In the simplest usage, the user just uploads the .gr format file from their hard drive and clicks ‘Submit’, and *structureMining* carries out the calculation and returns the result.

*structureMining* needs to know some composition information to do the calculation. If the file was generated using *PDFgetX2* (Qiu *et al.*, 2004 ▸), *PDFgetX3* (Juhás *et al.*, 2013 ▸), *PDFgetN* (Peterson *et al.*, 2000 ▸) or *PDFgetN3* (Juhás *et al.*, 2018 ▸) and the user entered the correct compositional information at the time of doing the data reduction to PDF, *structureMining* will find the information from the file header and use it. Files generated from other programs that contain a header with the metadata in the same form may be renamed as *.gr and used in the same way. In the future, we will also support other ways of delivering metadata such as composition.

If there are no compositional data in the header, or if they are incorrect, or if the user wants more control over the search heuristic that *structureMining@PDFitc* uses, the user can specify a chemical composition in the ‘Composition’ text box. At the time of writing, the available *structureMining* heuristics are:

(i) Search using exact composition. Type it as, for example, BaTiO3;

(ii) Search using a complete list of the constituent elements without specifying stoichiometry, for example, Ba-Ti-O;

(iii) Search using a subset of the constituents, with one additional wild-card constituent, *e.g.* Ba-O-*;

(iv) Search using a subset of the constituents with two additional wild-card constituents, *e.g.* Ba-*-*. This pattern can be extended for any number of constituents. This information can be found under the ‘Instructions’ link on the *structureMining@PDFitc* page.

**Figure 3:**
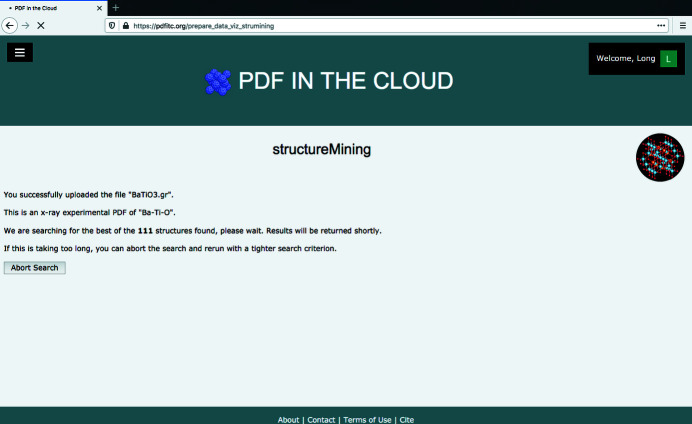
The intermediate waiting page after submitting a *structureMining* job.

Here we search for Ba-Ti-O as an example. After clicking the ‘Submit’ button, the user will be redirected to an intermediate page, as shown in Fig. 3 ▸, to wait for the *structureMining* job to be finished in the cloud. It lists the input data filename and the found or specified chemical composition. It shows the total number of structures meeting the heuristic from all the connected structural databases and has an abort button to be used if anything is wrong in the specification or the search is taking too long and needs to be run with a tighter heuristic.

Beyond composition, *structureMining* also takes a number of parameters it needs for the calculations from the file headers and, if it cannot find them, it uses reasonable defaults. *structureMining@PDFitc* allows the user to specify explicit values for any of these parameters in the ‘Optional Parameter’ text box. The most common one that the user may want to vary is the *r* range of the fit (default values of 1.5 < *r* < 20 Å being taken by default), for example, but it is also possible to specify *Q*_min_, *Q*_max_, and the instrument resolution parameters *Q*_damp_ and *Q*_broad_ *etc.* The user can click on ‘Instructions’ at *PDFitc* to see the syntax. This calculation job on 111 candidate structures took about 160 s to finish using two CPU cores on the cloud server, and could be easily speeded up by running on more cores.

**Figure 4:**
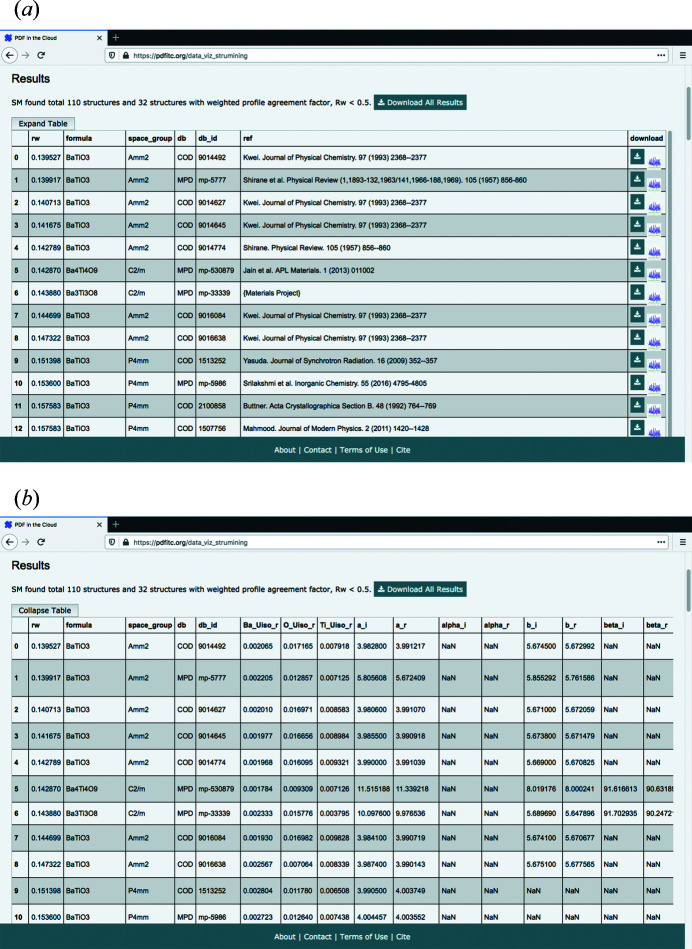
The *structureMining@PDFitc* result page showing (*a*) the compact and (*b*) the expanded table forms.

**Figure 5:**
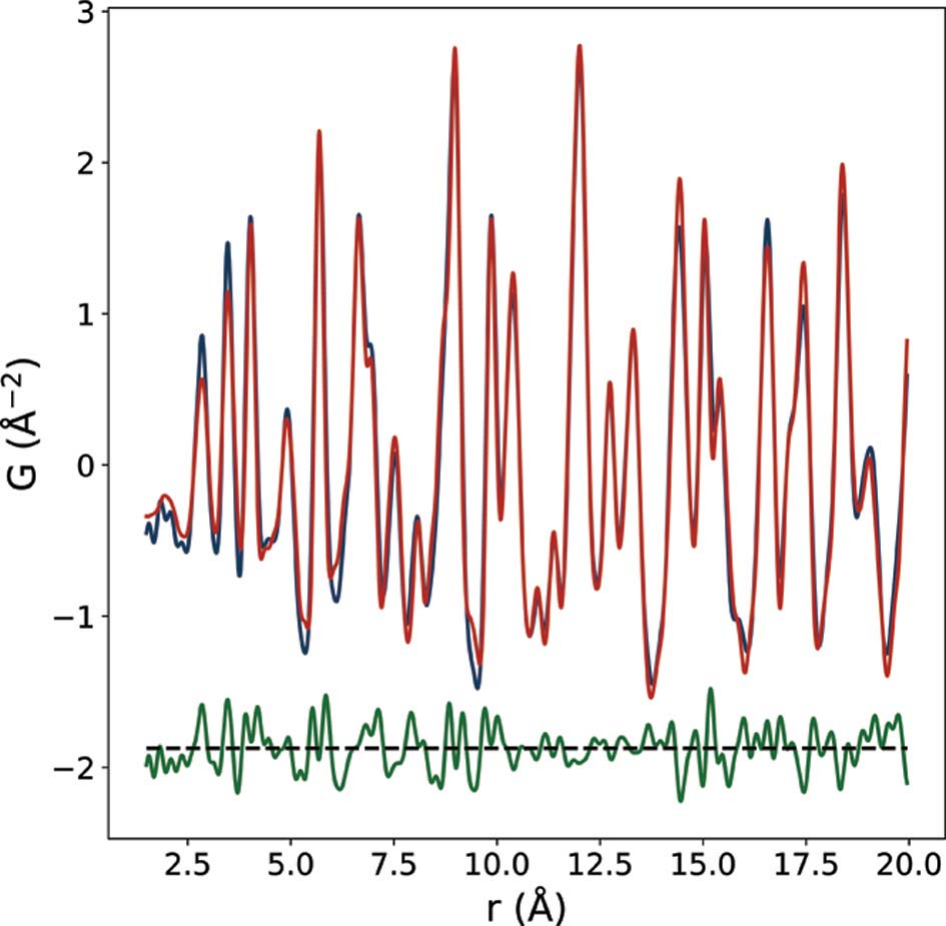
The PDF of barium titanate nanoparticles (blue curve) with the best-fit calculated PDF (red) for the top-ranked structure from *structureMining*. The difference curve is shown offset below in green.

When finished, *structureMining@PDFitc* returns a ‘Results’ table sorted by the goodness of fit *R*_*w*_ value which can be toggled to a compact or expanded form, as shown in Fig. 4 ▸. The compact table contains enough information to assess which structures the user may want to download for further study, with the expanded table additionally showing the most interesting starting and refined structural parameters for a slightly more in-depth review of the returned structures, such as the lattice parameters and the isotropic atomic displacement parameters for each element atom. In addition, an optional spherical particle diameter parameter can be refined if the PDF comes from nano-sized objects by having the experimenter specify an initial value (in units of ångströms). All the available optional structural parameters and their usage can be found in the ‘Instructions’ link at *structureMining@PDFitc*. There is also a figure icon [Fig. 4 ▸(*a*) at the right-hand end of each row of the table] that will display the fit when clicked, *e.g.* Fig. 5 ▸ is the fit for the top-ranked structure in our Ba-Ti-O search.

Users can then choose to download any of the found structures for further study. By clicking the download button they are provided with a zip file containing either database IDs of CIF files, or CIF files themselves (depending on licensing agreements with databases), of starting structures and refined structures, bibliographic references to the papers describing the original work where available,^1^ and a figure of the fit obtained by *structureMining*. A .csv file is also returned with all the initial and refined structural parameters from the *structureMining* fit.

For example, the top-ranked structure entry in our Ba-Ti-O example is the Crystallography Open Database (COD) (Gražulis *et al.*, 2009 ▸) ID 9014492 (Kwei *et al.*, 1993 ▸) BaTiO_3_ structure with space group *Amm*2. This structure was also found to be the best-fit structure to the PDFs from the gel-synthesized nanoparticles in the original work (Lombardi *et al.*, 2019 ▸).

Finally, by clicking the ‘Download All Results’ button, the user can download the results of all the structure fits found in the search for further investigation. We reiterate here that the main goal of *structureMining* is not to do high-quality fits, but to identify a set of candidate structures and return them for further study. It is highly likely that the quality of the fits may be improved by manual processing by the user after downloading.

## 4. Conclusions   

A community cloud web platform, called *PDF in the cloud* (*PDFitc*), that hosts applications for pair distribution function (PDF) analysis is available at https://www.pdfitc.org. It will host an increasing number of web services over time, but currently has programs that, given just a PDF, can discover a list of likely structural candidates and predict the most likely space group of the underlying structure. It also provides a program that calculates the similarity from a set of PDFs. The user can simply upload PDFs to one of the available analysis applications and get the answer back once the calculation finishes in the cloud. The structure-finding program, *structure­Mining*, was used as an example application here to demonstrate the straightforward simple workflow on *PDFitc*.

## Footnotes

1 Note that the Materials Project Database does not usually return the reference to the original work but self-references instead.
